# Prevalence of scabies and impetigo in the Solomon Islands: a school survey

**DOI:** 10.1186/s12879-019-4382-8

**Published:** 2019-09-13

**Authors:** Millicent H. Osti, Oliver Sokana, Sophie Phelan, Michael Marks, Margot J. Whitfeld, Christina Gorae, John M. Kaldor, Andrew C. Steer, Daniel Engelman

**Affiliations:** 1 Tropical Diseases Research Group, Murdoch Children’s Research Institute, Melbourne, Australia; 2 Department of Paediatrics, University of Melbourne, Melbourne, Australia; 3 Melbourne Children’s Global Health, Melbourne, Australia; 4 Ministry of Health and Medical Services, Honiara, Solomon Islands; 5 Kirby Institute, University of New South Wales, Sydney, Australia; 6 Research School of Population Health, Australian National University, Canberra, Australia; 7 Clinical Research Department, Faculty of Infectious and Tropical Diseases, London School of Hygiene and Tropical Medicine, London, UK; 8 St Vincent’s Hospital, Sydney, Australia; 9 Department of Medicine, University of New South Wales, Sydney, Australia

**Keywords:** Scabies, Impetigo, Diagnostic accuracy, Sarcoptes scabiei, Neglected tropical diseases

## Abstract

**Background:**

Scabies, a parasitic disease of the skin, is a major public health problem, largely affecting children. Scabies is often complicated by impetigo which can result in serious complications including invasive infections and immune mediated diseases. Scabies and impetigo are reported to have high prevalence in tropical settings including the Solomon Islands.

**Methods:**

We conducted a cross-sectional prevalence survey at Gizo Primary School in the Western Province of the Solomon Islands in August 2018. The diagnosis of scabies was based on criteria developed by the International Alliance for the Control of Scabies in 2018. Population attributable risk was calculated to determine the effect of scabies on the prevalence of impetigo, and both adjusted and unadjusted risk ratios were calculated to identify differences between sexes and age groups.

**Results:**

A total of 324 students were assessed (47.5% of those enrolled at the school). The prevalence of scabies was 54.3% (95% confidence interval [CI] 48.7–59.8) and most disease was mild (68.8%). The prevalence was higher in males (63.5%; adjusted risk ratio [ARR] 1.4, 95% CI 1.1–1.7), and in those aged 10–12 years (61.4%; ARR 1.8, 95% CI 1.1–2.9 when compared to those aged 4–6 years). The prevalence of impetigo was 32.1%, with males more likely to be affected (41.7%, ARR 1.7, 95% CI 1.2–2.4) but with no significant differences between age groups. 63.5% of those with impetigo had scabies, corresponding to a population attributable risk of 11.8%.

**Conclusions:**

There is a very high burden of scabies and impetigo among primary school students in Gizo. There is a critical need for the development and implementation of control programs in areas where scabies is endemic.

## Background

*Sarcoptes scabiei* var. *hominis* is a parasitic mite that burrows into human skin. Infestation with the mite results in an inflammatory reaction producing skin lesions with severe pruritus. Persistent scratching results in broken skin, compromising its barrier function and facilitating secondary bacterial infection with group A *Streptococcus* (GAS; *Streptococcus pyogenes*) or with *Staphylococcus aureus*, presenting as impetigo*.* Impetigo may progress to severe skin and soft tissue infection, bone and joint infection, sepsis or the immune-mediated complications of GAS infection such as glomerulonephritis and potentially rheumatic fever [1, 2]. Although a range of effective treatment options for scabies exists, the highly contagious nature of the disease makes its public health control challenging.

Scabies is estimated to affect more than 200 million people, especially children [3, 4]. Despite these estimates, a major barrier for global disease control is the scarcity of epidemiologic data [5, 6]. Prevalence surveys using systematic and standardized methods are likely to be a crucial component in determining the global burden of disease and identifying areas where the disease is endemic. The International Alliance for the Control of Scabies (IACS) undertook a Delphi study in order to establish consensus diagnostic criteria for scabies [7]. The 2018 IACS Criteria provide standardized diagnostic methods to enable evaluation and comparison of global prevalence data.

Schools have been a focus for both surveillance and control of neglected tropical diseases (NTDs), including schistosomiasis and soil-transmitted helminthiases [8]. School screening studies conducted in Africa and Asia have investigated the burden of scabies, either in isolation, or in conjunction with other NTDs or skin conditions [9, 10] but there have been no school-based studies from the Solomon Islands. Locally relevant epidemiological evidence of the burden of scabies is required for the design and implementation of community scabies control programs. The primary objective of this study was therefore to determine the prevalence of scabies and impetigo in the population examined using a school screening method. The secondary objectives were to investigate features associated with scabies and impetigo. We also aimed to describe the severity of scabies and impetigo and the distribution of scabies cases using the 2018 IACS Criteria subcategories.

## Methods

### Study setting and participants

This was a cross-sectional, school-based study that took place in the public primary school in the town of Gizo, the capital of the Western Province of the Solomon Islands. The population in the Gizo region is approximately 7000 [11]. The setting was selected because it is the site of a planned trial. The school was selected as the study site because of the simple facilitation of recruitment of participants within a school setting and its convenient location within the centre of the town.

At the commencement of 2018, the primary school had a school roll of 682 students, consisting of students residing in Gizo town and those who travel by truck or boat from surrounding villages or islands. Students at the primary school are aged 4 to 15 years, with class groupings based on aptitude rather than age.

### Study procedures

All students attending Gizo Primary School were eligible for inclusion in the study. All patients underwent a standardized examination for scabies and impetigo.

Clinical assessment was performed by doctors with expertise in scabies diagnosis, and by nurses with clinical experience in scabies diagnosis, supplemented by a training program on the diagnosis of scabies, impetigo and other locally common skin conditions. Scabies was diagnosed using the 2018 IACS Criteria [7]. Although eight subcategories exist in the IACS Criteria, only four were relevant for diagnosis in this study (Table 1). Subcategories of diagnosis that required specialized equipment (including microscopy and dermoscopy) or required examination of the genitals, were not used, as these were not practical for a field survey in this context. Impetigo was diagnosed on the presence of papules, pustules or ulcerative lesions with associated erythema, crusting or pus. Skin examination consisted of assessment of exposed areas: the feet and legs to the thighs, hands to the upper arms, neck, face and scalp. Students were in school uniform which consisted of above-knee shorts and above-elbow shirts or dresses. Shoes were removed prior to examination. A focused history of standardized questions was taken of all participants with skin lesions consisting of information required for the IACS Criteria classification. Questions included whether participants experienced itch. Contact history was assessed by asking if participants lived with someone, or had a friend or classmate with itch, or if they lived with someone, or had a friend or classmate with a rash that looks like scabies. Participants were shown images of people with typical scabies rashes to assist these questions. History was taken in the local language (Solomon Islands Pijin). Permethrin was supplied for individuals diagnosed with scabies. Permethrin was also supplied for household contacts of diagnosed children, as per local treatment protocol. No treatment was supplied to examined children without scabies. If other conditions were diagnosed, referral was provided to the local health clinic.

**Table 1:** Case definitions for scabies using the 2018 IACS Criteria

Criteria Category		Used in Survey
*Confirmed scabies*		
A1:	Mites, eggs or feces on light microscopy of skin samples	**No**
A2:	Mites, eggs or feces visualized on individual using high powered imaging device	**No**
A3:	Mite visualized on individual using dermoscopy	**No**
*Clinical Scabies*		
B1:	Presence of burrows	Yes*
B2:	Typical lesions affecting male genitalia	**No**
B3:	(Typical lesions) and (typical distribution) and (itch) and (contact history)	**Yes**
*Suspected Scabies*		
C1:	(Typical lesions) and (typical distribution) and (itch) or (contact history)	**Yes**
C2:	(Atypical lesions) or (atypical distribution) and (itch) and (contact history)	**Yes**

* If burrows were identified, individuals were also classified as either B3, C1, or C2, as these were not confirmed with dermoscopy

*A diagnosis of scabies should only be made if other differential diagnoses are considered less likely than scabies.*

### Data collection and statistical methods

Demographic information was collected including participant age, sex and school grade.

Scabies diagnosis was classified into one of three IACS Criteria subcategories (Table 1).

Following methods used in previous studies, severity of scabies and impetigo was assessed based on the number of lesions present [12, 13]. Scabies was categorized as: mild, 1 to 10 lesions; moderate, 11 to 49 lesions; or severe, 50 or more lesions. Impetigo was classified as: very mild, 1 to 5 lesions; mild, 6 to 10 lesions; moderate, 11 to 49 lesions; or severe, 50 or more lesions.

Prevalence for scabies and impetigo was calculated for each demographic group. Risk ratios were calculated using generalized linear models, adjusting for age group and sex. Population attributable risk was calculated to further analyze association between scabies and impetigo [14].

Data were collected on forms during the study period and then transcribed and uploaded to a REDCap study database (Research Electronic Data Capture), hosted at Murdoch Children’s Research Institute [15]. All statistical analysis was performed using Stata (version 15, StataCorp, College Station, TX, USA).

### Ethics statement

Parents were provided with written information regarding the study. A community information night was also held at the school where members of the study team provided further information and answered questions. Written consent was obtained from parents or guardians of all participants and verbal assent from children where possible.

Ethical approval was granted by the Royal Children’s Hospital Melbourne Human Research Ethics Committee (reference number 38099A) and the Solomon Islands Health Research and Ethics Review Board (reference number 05/18).

## Results

Over 6 days in August 2018, 324 students were recruited and examined (47.5% of those enrolled at the school). All consenting students present at school during study days were examined. Doctors examined 247 participants (76.2%) and the remainder were examined by trained nurse examiners. Of those examined, 51.9% were female and the mean age was 9.6 years (standard deviation 2.3, range 4–15; Table 2).

**Table 2:** Participant demographics and prevalence of scabies and impetigo (*N* = 324)

Study Sample		Scabies			Impetigo		
	N (%)	n	% (95%CI)	Adjusted RR (95%CI)	n	% (95%CI)	Adjusted RR (95%CI)
Sex							
Female	168 (51.9)	77	45.8 (38.1-53.7)	Ref	39	23.2 (17.1-30.3)	Ref
Male	156 (48.2)	99	63.5 (55.4-71.0)	1.4 (1.1-1.7)	65	41.7 (33.8-49.8)	1.7 (1.2-2.4)
Age (Years)							
4-6	35 (10.8)	12	34.3 (19.1-52.2)	Ref	14	40.0 (23.9-57.9)	Ref
7-9	121 (37.4)	66	54.5 (45.2-63.6)	1.6 (1.0-2.6)	44	36.4 (27.8-45.6)	1 (0.6-1.5)
10-12	132 (40.7)	81	61.4 (52.5-69.7)	1.8 (1.1-2.9)	35	26.5 (19.2-34.9)	0.7 (0.5-1.2)
13-15	36 (11.1)	17	47.2 (30.4-64.5)	1.4 (0.8-2.5)	11	30.6 (16.3-48.1)	0.8 (0.4-1.5)

### Scabies and impetigo prevalence

Overall, 176 participants had scabies (prevalence 54.3, 95% confidence interval [CI] 48.7–59.8), and 104 participants had impetigo (prevalence 32.1, 95% CI 27.0–37.5). Most scabies cases were mild (68.8%, *n* = 121) but 14 participants (8%) had severe scabies (Table 3). The majority of those with impetigo had very mild disease (*n* = 86, 82.7%, Table 3). The prevalence of scabies diagnosed by doctor and nurse examiners was similar (52.2, 95% CI 45.8–58.6; vs 61.0, 95% CI 49.2–72.0%).

**Table 3:** Details of diagnosis and severity for scabies and impetigo

	Overall (*N* = 324)	Male (*N* = 156)	Female (*N* = 168)	Age Years 4–6 (*N* = 35)	Age Years 7–9 (*N* = 121)	Age Years 10–12 (*N* = 132)	Age Years 13–15 (*N* = 36)
Scabies	n (%)	n (%)	n (%)	n (%)	n (%)	n (%)	n (%)
Prevalence	176 (54.3)	99 (63.5)	77 (45.8)	12 (34.3)	66 (54.6)	81 (61.4)	17 (47.2)
*Category*							
B1: Burrows*	4 (2.3)	2 (2)	2 (2.6)	0 (0)	0 (0)	3 (3.7)	1 (5.9)
B3: Clinical scabies	128 (72.7)	73 (73.7)	55 (71.4)	9 (75)	47 (71.2)	64 (79)	8 (47.1)
C1: Suspected scabies	41 (23.3)	25 (25.2)	16 (20.8)	3 (25)	16 (24.2)	13 (19.7)	9 (52.9)
C2: Suspected scabies	7 (4)	1 (1)	6 (7.8)	0 (0)	3 (4.5)	4 (6.1)	0 (0)
*Severity*							
Mild (1–10)	121 (68.8)	67 (67.7)	54 (70.1)	8 (66.7)	46 (69.7)	53 (65.4)	14 (82.4)
Moderate (11–49)	41 (23.3)	22 (22.2)	19 (24.7)	4 (33.3)	15 (22.7)	20 (24.7)	2 (11.8)
Severe (≥ 50)	14 (8)	10 (10.1)	4 (5.2)	0 (0)	5 (7.6)	8 (9.9)	1 (5.9)
Impetigo	n (%)	n (%)	n (%)	n (%)	n (%)	n (%)	n (%)
Prevalence	104 (32.1)	65 (41.7)	39 (23.2)	14 (40)	44 (36.4)	35 (26.5)	11 (30.6)
*Severity*							
Very Mild (≥ 5)	86 (82.7)	52 (80.0)	34 (87.2)	12 (85.7)	39 (88.6)	25 (71.4)	10 (90.9)
Mild (6–10)	12 (11.5)	8 (12.3)	4 (10.3)	2 (14.3)	4 (9.1)	6 (17.1)	0 (0)
Moderate (11–49)	3 (2.9)	2 (3.1)	1 (2.6)	0 (0)	0 (0)	2 (5.7)	1 (9.1)
Severe (≥50)	3 (2.9)	3 (4.6)	0 (0)	0 (0)	1 (2.3)	2 (5.7)	0 (0)

*Individuals with burrows were additionally categorized as either B3, C1 or C2

### History features

Itch was reported in 81.8% of individuals with scabies (*n* = 144) and in 18.4% of those without scabies (*n* = 21, missing = 34). A positive contact history was reported in 92.1% (*n* = 161) of those with scabies, and in 52.6% of those without scabies who were asked (*n* = 60, missing = 34). The most common contact history reported was having a friend or classmate with a rash that looks like scabies (75, 95%CI 67.9–81.2, Table 4). Of those with scabies, 26.1% (*n* = 46) reported either a history of itch or a positive contact history and 73.9% (*n* = 130) had both history features. Of those without scabies, 46.5% (*n* = 53) reported either a history of itch or a positive contact history and 12.3% (*n* = 14) had both features.

**Table 4:** Reporting of history features

	Total (*N* = 290)		With scabies (*N* = 176)		Without scabies (*N* = 114)	
Itch	n	% (95%CI)	n	% (95%CI)	n	% (95%CI)
Itch present	165	56.9 (51–62.7)	144	81.8 (75.3–87.2)	21	18.4 (11.8–26.8)
Contact History						
House contact with itch	97	33.5 (28–39.2)	76	43.2 (35.8–50.8)	21	18.4 (11.8–26.8)
School contact with itch	152	52.4 (46.5–58.3)	117	66.5 (59–73.4)	35	30.7 (22.4–40)
House contact with scabies rash	76	27.2 (22.2–32.8)	65	36.9 (29.8–44.5)	14	12.3 (6.9–19.7)
School contact with scabies rash	177	61 (55.2–66.7)	132	75 (67.9–81.2)	45	39.5 (30.4–49.1)
Contact history positive*	222	76.6 (71.2–81.3)	162	92.1 (87–95.5)	60	52.6 (43.1–62.1)

*Contact history was considered positive if responses to any of the four questions were positive

### IACS criteria

The majority of scabies cases (*n* = 128, 72.7%) were classified as Clinical Scabies, subcategory B3, with typical clinical features, itch and contact history according to the IACS Criteria (Table 3). Only four individuals (2.3%) were identified to have clinically visible burrows (B1).

### Associations with scabies and impetigo

**Fig. 1:**
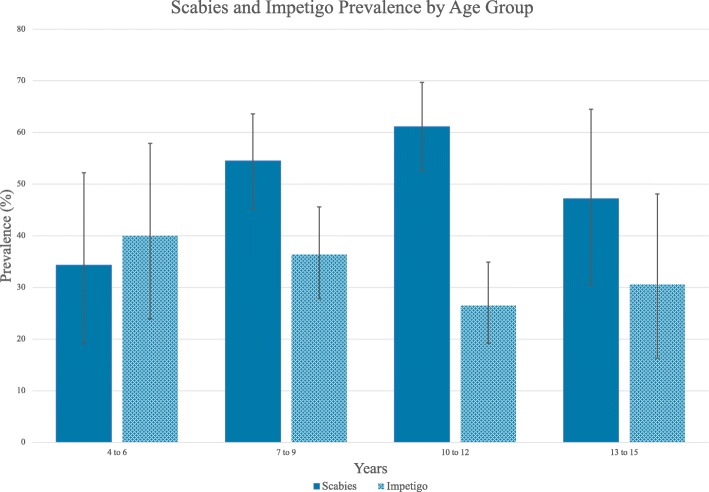
Prevalence of scabies and impetigo by age group with 95% confidence intervals

Scabies was more common in males than females (63.5% vs 45.8%, adjusted risk ratio [ARR] 1.4, 95% CI 1.1–1.7; Table 2) and the most commonly affected age group was children aged 10–12 years (61.4%; ARR 1.8, 95% CI 1.1–2.9, compared to age group 4–6 years; Fig. 1). Prevalence of impetigo was higher in males (41.7% vs 23.2%, ARR 1.7, 95% CI 1.2–2.4) but similar across age groups.

Participants with scabies had a higher prevalence of impetigo than those without scabies (66 of 176, 37.5% vs 38 of 110, 25.7%, ARR 1.5, 95% CI 1.1–2). Overall, 63.5% of those with impetigo had scabies, corresponding to a population attributable risk of 11.8% (95%CI 1.7–21.7, *P* = 0.02). Of the 18 individuals with greater than very mild impetigo, 14 (77.8%) had scabies, and of the individuals with moderate to severe impetigo, all had scabies (*n* = 6, 100%).

## Discussion

Our study highlights the very high prevalence of scabies (54%) and impetigo (32%) in school-aged children in Gizo. The prevalence of scabies is higher than a previous study conducted in the same region in 2014 which reported a prevalence of 23% in children aged 5 to 14 [12]. High scabies prevalence has been described in other disadvantaged populations including in children in Fiji (55.7%) [13], rural communities in Nigeria (65%) [16] and amongst asylum seekers in the Netherlands (33.5%) [17].

In our study, males were more likely than females to have scabies, which has been reported previously [18, 19], although this finding is not represented in the Global Burden of Disease Study (2015) estimates [20].

**Fig. 2:**
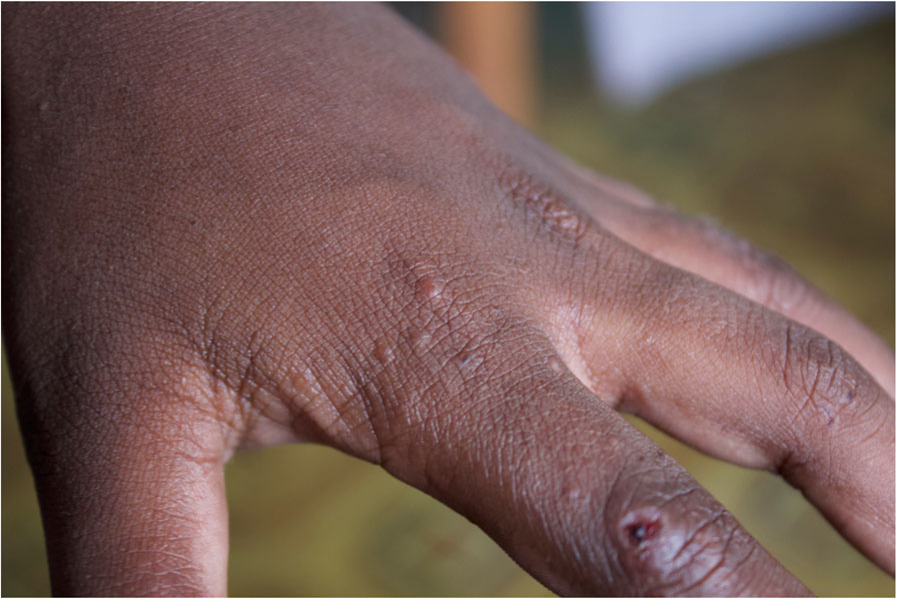
Typical scabies lesions on a child’s fingers

The majority of scabies seen was classified as Clinical Scabies according to the 2018 IACS Criteria (Fig. 2). This was consistent for males and females. Very few scabies cases (4%) had either atypical lesions or an atypical distribution of their rash. We found that the 2018 IACS Criteria for the diagnosis of scabies were easily integrated and implemented into our study design. Implementation of these criteria in other settings would allow comparison across studies and regions.

The high prevalence of impetigo (32%) is consistent with other studies from Pacific Island nations, including in Fiji (26%) and a previous study in the Solomon Islands (33%) [12, 21]. Most of the impetigo (82%) in this study was very mild, consistent with other studies [12, 13]. Impetigo was positively associated with the presence of scabies, although the association was less strong than previously reported [13]. The association between scabies and impetigo in a range of settings warrants further investigation.

Itch was reported by 82% of participants with scabies, which is consistent with other studies (72–100%) [16, 17, 22–26]. A positive contact history was reported in 92% of participants with scabies. In those with scabies, itch was reported to occur in 43% of household members, similar to other studies (50–55%) [22, 24]. However, in our study, more children identified contact with a friend or class member than a household member. Contact between children at school and between friends is likely to be important for disease transmission between children. Contact history questions should be asked in an appropriate way for specific survey settings and participants.

There were several limitations to our study. First, this study only focused on school children and is therefore not generalizable to other age groups. However, as scabies is highly transmissible among household contacts [27] it is likely that there would be a high prevalence amongst the household members of children with scabies. Second, we enrolled 47.5% of students on the school roll, which may have introduced participation bias. The remaining students did not participate because they were away from school or did not provide consent to participate. Those not attending school may have been more disadvantaged with an even greater disease burden, possibly leading to an underestimate of the true prevalence. Conversely, it is possible that some families and teachers may have encouraged children with symptoms of scabies or impetigo to participate, which may have led to an overestimate of prevalence. Third, the diagnosis of scabies and impetigo in the study was based on clinical assessment of exposed areas utilizing the IACS Criteria, rather than whole body examination using dermoscopy, microscopy, or skin scrapings. Limited examination may have underestimated the true prevalence, perhaps by up to 10% [28], whereas reliance on clinical signs may have led to some misclassification and overestimate of disease.

Children in low-resource settings are disproportionately affected by scabies and impetigo, predisposing them to significant morbidity and mortality. The high prevalence of scabies in this study supports the ongoing need for prevalence studies and identifying communities where the disease is endemic. The significant burden of disease highlights the need for ongoing health promotion activities for scabies and impetigo. The very high prevalence of scabies also supports the urgent implementation of a population-scale control program in the Solomon Islands. Greater appreciation of scabies as a major global contributor to poor health in children may help to improve awareness and access to treatment in settings where scabies is endemic.

## Data Availability

The datasets used and analyzed during the study are available from the corresponding author on reasonable request.

## Abbreviations

ARR: Adjusted risk ratio
CI: Confidence interval
GAS: Group A *Streptococcus*
IACS: International Alliance for the Control of Scabies
NTDs**-**: Neglected Tropical Diseases

## References

[1] Lawrence G, Leafasia J, Sheridan J, Hills S, Wate J, Wate C (2005). Control of scabies, skin sores and haematuria in children in the Solomon Islands: another role for ivermectin. Bull World Health Organ.

[2] Thornley S, Marshall R, Jarrett P, Sundborn G, Reynolds E, Schofield G (2018). Scabies is strongly associated with acute rheumatic fever in a cohort study of Auckland children. J Paediatr Child Health.

[3] Romani L, Steer AC, Whitfeld MJ, Kaldor JM (2015). Prevalence of scabies and impetigo worldwide: a systematic review. Lancet Infect Dis.

[4] Vos T, Allen C, Arora M, Barber RM, Bhutta ZA, Brown A (2016). Global, regional, and national incidence, prevalence, and years lived with disability for 310 diseases and injuries, 1990–2015: a systematic analysis for the Global Burden of Disease Study 2015. Lancet.

[5] WHO. Report of the tenth meeting of the WHO Strategic and Technical Advisory Group for neglected tropical diseases. Geneva: WHO; 2017. Available at: https://www.who.int/neglected_diseases/NTD_STAG_report_2017.pdf.

[6] Engelman D, Steer AC (2018). Control strategies for scabies. Trop Med Infect Dis.

[7] Engelman D, Fuller LC, Steer AC (2018). International Alliance for the control of scabies Delphi p. Consensus criteria for the diagnosis of scabies: a Delphi study of international experts. PLoS Negl Trop Dis.

[8] WHO. Helminth control in school-age children: a guide for managers of control programmes. Second ed. Geneva: World Health Organization; 2012.

[9] Yotsu RR, Kouadio K, Vagamon B, N'Guessan K, Akpa AJ, Yao A (2018). Skin disease prevalence study in schoolchildren in rural cote d'Ivoire: implications for integration of neglected skin diseases (skin NTDs). PLoS Negl Trop Dis.

[10] Korte LM, Bowen AC, Draper ADK, Davis K, Steel A, Teodora I (2018). Scabies and impetigo in Timor-Leste: a school screening study in two districts. PLoS Negl Trop Dis.

[11] Solomon Islands Government. Provincial profile of the 2009 Population & Housing Census: Western. Honiara: Solomon Islands Government; 2009.

[12] Mason DS, Marks M, Sokana O, Solomon AW, Mabey DC, Romani L (2016). The prevalence of scabies and impetigo in the Solomon Islands: a population-based survey. PLoS Negl Trop Dis.

[13] Romani L, Whitfeld MJ, Koroivueta J, Kama M, Wand H, Tikoduadua L (2017). The epidemiology of scabies and impetigo in relation to demographic and residential characteristics: Basline findings from the skin health intervention Fiji trial. Am J Trop Med Hyg.

[14] Newson RB (2013). Attributable and unattributable risks and fractions and other scenario comparisons. Stata J.

[15] Harris PA, Taylor R, Thielke R, Payne J, Gonzalez N, Conde JG (2009). Research electronic data capture (REDCap) - a metadata-driven methodology and workflow process for providing translational research infomatics support. J Biomed Inform.

[16] Ugbomoiko US, Oyedeji SA, Babamale OA, Heukelbach J (2018). Scabies in resource-poor communities in Nasarawa state, Nigeria: epidemiology, clinical features and factors associated with infestation. Trop Med Infect Dis.

[17] Beeres DT, Ravensbergen SJ, Heidema A, Cornish D, Vonk M, Wijnholds LD (2018). Efficacy of ivermectin mass-drug administration to control scabies in asylum seekers in the Netherlands: a retrospective cohort study between January 2014 - march 2016. PLoS Negl Trop Dis.

[18] Anderson KL, Strowd LC (2017). Epidemiology, diagnosis, and treatment of scabies in a dermatology Office. J Am Board of Fam Med.

[19] Kouotou EA, Nansseu JR, Kouawa MK, Zoung-Kanyi Bissek AC (2016). Prevalence and drivers of human scabies among children and adolescents living and studying in Cameroonian boarding schools. Parasite Vectors.

[20] Karimkhani C, Colombara DV, Drucker AM, Norton SA, Hay R, Engelman D (2017). The global burden of scabies: a cross-sectional analysis from the global burden of disease study 2015. Lancet Infect Dis.

[21] Steer AC, Jenney AW, Kado J, Batzloff MR, La Vincente S, Waqatakirewa L (2009). High burden of impetigo and scabies in a tropical country. PLoS Negl Trop Dis.

[22] Dupuy A, Dehen L, Bourrat E, Lacroix C, Benderdouche M, Dubertret L (2007). Accuracy of standard dermoscopy for diagnosing scabies. J Am Acad Dermatol.

[23] Jackson A, Heukelbach J, Filho AF, Junior Ede B, Feldmeier H (2007). Clinical features and associated morbidity of scabies in a rural community in Alagoas, Brazil. Tropical Med Int Health.

[24] Boralevi F, Diallo A, Miquel J, Guerin-Moreau M, Bessis D, Chiaverini C (2014). Clinical phenotype of scabies by age. Pediatrics.

[25] Nair PA, Vora RV, Jivani NB, Gandhi SS (2016). A study of clinical profile and quality of life in patients with scabies at a rural tertiary care Centre. J Clin Diagn Res.

[26] Worth C, Heukelbach J, Fengler G, Walter B, Liesenfeld O, Feldmeier H (2012). Impaired quality of life in adults and children with scabies from an impoverished community in Brazil. Int J Dermatol.

[27] Hay RJ, Steer AC, Engelman D, Walton S (2012). Scabies in the developing world--its prevalence, complications, and management. Clin Microbiol Infect.

[28] Marks M, Engelman D, Romani L, Mason D, Sokana O, Kama M (2018). Exploration of a simplified clinical examination for scabies to support public health decision-making. PLoS Negl Trop Dis.

